# Predicting EQ-5D full health state in systemic lupus erythematosus using machine learning algorithms

**DOI:** 10.1093/rap/rkaf032

**Published:** 2025-04-18

**Authors:** João Botto, Nursen Cetrez, Dionysis Nikolopoulos, Malin Regardt, Emelie Heintz, Julius Lindblom, Ioannis Parodis

**Affiliations:** Division of Rheumatology, Department of Medicine Solna, Karolinska Institutet, Stockholm, Sweden; Department of Gastroenterology, Dermatology and Rheumatology, Karolinska University Hospital, Stockholm, Sweden; Division of Rheumatology, Department of Medicine Solna, Karolinska Institutet, Stockholm, Sweden; Department of Gastroenterology, Dermatology and Rheumatology, Karolinska University Hospital, Stockholm, Sweden; Division of Rheumatology, Department of Medicine Solna, Karolinska Institutet, Stockholm, Sweden; Department of Gastroenterology, Dermatology and Rheumatology, Karolinska University Hospital, Stockholm, Sweden; Department of Neurobiology, Care Sciences and Society, Division of Occupational Therapy, Karolinska Institutet, Stockholm, Sweden; Women’s Health and Allied Health Professionals Theme, Medical Unit Occupational Therapy and Physiotherapy, Karolinska University Hospital, Stockholm, Sweden; Department of Learning, Informatics, Management and Ethics (LIME), Karolinska Institutet, Stockholm, Sweden; Division of Rheumatology, Department of Medicine Solna, Karolinska Institutet, Stockholm, Sweden; Department of Gastroenterology, Dermatology and Rheumatology, Karolinska University Hospital, Stockholm, Sweden; Division of Rheumatology, Department of Medicine Solna, Karolinska Institutet, Stockholm, Sweden; Department of Gastroenterology, Dermatology and Rheumatology, Karolinska University Hospital, Stockholm, Sweden; Department of Rheumatology, Faculty of Medicine and Health, Örebro University, Örebro, Sweden

**Keywords:** systemic lupus erythematosus, patient-reported outcomes, quality of life, EQ-5D, machine learning

## Abstract

**Objectives:**

To determine factors associated with reports of EuroQol 5-Dimensions (EQ-5D) full health state (FHS) before and after a trial intervention in patients with SLE, resorting to machine learning algorithms.

**Methods:**

We conducted a post hoc analysis of two phase 3 clinical trials of belimumab (BLISS-52, BLISS-76). Demographic, laboratory and clinical features were retrieved and the Monte Carlo Feature Selection algorithm was employed, then further refined upon consideration of collinearity and clinical relevance. We used support vector machine with radial basis function kernel (SVMRadial), least absolute shrinkage and selection operator (LASSO), neural network (NNet) and logistic regression (LR) to capture both linear and non-linear relationships while ensuring interpretability and robustness.

**Results:**

Among 1642 SLE patients, 12.9% reported FHS at baseline and 23.1% at week 52. Selected features were age, sex, Asian ancestry, baseline cSLEDAI-2K, SELENA-SLEDAI PGA, and urine protein:creatinine ratio (UPCR) and baseline EQ-5D 3-Levels (EQ-5D-3L) index score (week 52 models only). The models predicting FHS demonstrated comparable performance at baseline and week 52. A maximum area under the curve of 0.73 was seen for the baseline LASSO and LR models and a maximum of 0.77 for the week 52 LASSO and NNet models. Negative predictive values were high for all models (0.88–0.94). Calibration showed marginal improvement in week 52 models.

**Conclusion:**

Machine learning identified older age, female sex, non-Asian ancestry, high disease activity and low UPCR to be associated with a lack of FHS experience in SLE patients at baseline and week 52. High baseline EQ-5D-3L index scores constituted the strongest predictor of FHS at week 52.

**Trial registration:**

The BLISS-52 and BLISS-76 trials are registered at www.clinicaltrials.gov (NCT00424476 and NCT00410384, respectively).

Key messages

- We used machine learning to determine factors associated with the EQ-5D full health state in SLE.
- Older age, female sex, non-Asian ancestry, high disease activity and low UPCR predicted non-FHS.
- High baseline EQ-5D-3L index scores constituted the main predictor of FHS experience at week 52.

## Introduction

SLE is a complex autoimmune disease that can cause inflammation and damage to multiple organ systems. It predominantly affects women of childbearing age and poses a significant burden on patients’ lives [1, 2]. In the assessment of this burden, patients’ health-related quality of life (HRQoL) constitutes an important parameter. Patients with SLE often experience significantly lower HRQoL compared with healthy individuals [3] and those with other chronic diseases [4]. Researchers have strived to understand the impact of SLE on HRQoL and which factors are associated with its variations [3, 4]. It is important to know that a good clinical response does not always correspond to a favourable HRQoL outcome [5]. Therefore, evaluating HRQoL has become increasingly important in SLE research and clinical practice [6, 7] and was proposed as one of the principal outcome domains proclaimed during the Outcome Measures in Rheumatology (OMERACT) IV consensus conference [8].

The EuroQol 5-Dimensions (EQ-5D) health questionnaire is widely used for measuring HRQoL [9] and its psychometric properties have been validated in SLE [10]. The EQ-5D consists of a visual analogue scale (VAS) for self-assessing health state on a scale ranging from 0 (worst possible health state) to 100 (best possible health state) and a descriptive system comprising five dimensions: mobility, self-care, usual activities, pain/discomfort and anxiety/depression. Each dimension is represented by one question, with three levels of severity in the EQ-5D-3L version: no problems (level 1), some problems (level 2) and extreme problems (level 3). The responses are put together to form an individual health profile (e.g. 21322) and can be converted into a single index score using preference-based population-specific scoring algorithms. This overall score can range from <0 (where 0 represents experience equal to death) to 1. A score of ‘no problems’ in all five dimensions yields an index score of 1, corresponding to an idealized state of complete health, which is termed the full health state (FHS) [11].

While therapeutic advancements have been made in the management of SLE [12], little is known about the factors that influence a patient’s ability to achieve and maintain an EQ-5D FHS. Identification of variables with the ability to predict attainment of an EQ-5D FHS in SLE could provide clinicians with more realistic expectations and with valuable insights for individualized patient care and optimization of the allocation of healthcare resources.

In a recent study, we reported an EQ-5D FHS prevalence of 23% among SLE patients from a clinical trial setting at week 52 from baseline and disclosed factors such as female sex, increasing BMI and non-Asian ethnicity to be negatively associated with an EQ-5D FHS at week 52 [13]. The present study aimed to determine factors that are associated with an EQ-5D FHS experience or its absence in patients with SLE from the same clinical trial setting, both at baseline and after 52 weeks from commencement of the trial intervention. This article pioneers the application of machine learning algorithms for addressing this question, which constitutes a novel approach in this context.

## Methods

### Study population

Data analysis was conducted using information from two phase 3 clinical trials assessing the efficacy of belimumab, a monoclonal antibody that hampers B cell survival and proliferation by binding to soluble B cell activating factor (BAFF), in patients with active SLE [BLISS-52 (NCT00424476; *n *=* *865) [14] and BLISS-76 (NCT00410384; *n *=* *819)] [15]. All individuals enrolled in these trials satisfied the revised classification criteria for SLE established by the ACR [16], were adults and had an ANA titre ≥1:80 and/or a serum anti-dsDNA antibody level ≥30 IU/ml at screening. Additionally, they exhibited a Safety of Estrogens in Lupus National Assessment–SLE Disease Activity Index (SELENA-SLEDAI) [17] score ≥6 at screening. All participants had been on a stable non-biologic standard therapy for at least 30 days prior to the trial baseline. Patients were not eligible for participation if they had active severe LN or CNS involvement.

The study participants were randomly assigned to one of three arms: belimumab 1 mg/kg, belimumab 10 mg/kg or placebo, administered intravenously, on top of standard therapy. The initial doses were given at weeks 0, 2 and 4 and were followed by subsequent doses every fourth week [14, 15].

Data from the BLISS trials were provided by GSK (Uxbridge, UK) through the Clinical Study Data Request consortium. Forty-two patients were excluded due to missing primary outcome data (EQ-5D FHS at baseline or week 52), resulting in a study population of 1642 SLE patients, to ensure robust outcome assessments.

### Ethics

All study participants provided written informed consent. The trial protocols received approval from regional ethics review boards across all participating centres. The protocol of the present analysis received approval from the Swedish Ethical Review Authority (2019-05498). The BLISS study protocols and the protocol of the present investigation adhered to the ethical guidelines outlined in the Declaration of Helsinki.

### Measures of disease activity and damage

The SLEDAI evaluates overall SLE activity using 24 descriptors, each assigned a specific weight based on its relative significance [18]. The SLEDAI-2000 (SLEDAI-2K) modification allows ongoing disease activity in the descriptors alopecia, mucosal ulcers, proteinuria and rash to be scored [19], unlike the original SLEDAI where only new occurrences are scored [18]. The clinical version of SLEDAI-2K (cSLEDAI-2K), which omits the serological descriptors (anti-dsDNA and complement) [20], was the one employed for assessing clinical disease activity in the present investigation.

The SELENA-SLEDAI physician global assessment (PGA) captures overall SLE activity on a scale ranging from 0 (none) to 3 (severe) [21].

Lastly, the Systemic Lupus International Collaborating Clinics (SLICC)/ACR damage index (SDI) measures irreversible organ damage that occurs from the time of SLE diagnosis onwards, irrespective of whether the damage is directly linked to SLE. The SDI comprises 39 items, grouped into 12 organ systems [22].

### Study outcome

The outcome in this study was a patient-reported FHS (*vs* non-FHS) assessed using the EQ-5D-3L instrument at baseline and at week 52. A FHS was defined as a report of no problems in all dimensions of the descriptive system of the EQ-5D-3L (health profile 11111).

### Data collection

Demographic parameters, baseline clinical and laboratory features and concomitant medications were retrieved. EQ-5D data were retrieved from the baseline and week 52 visits. EQ-5D-3L index scores were calculated based on the US population scoring algorithm [23].

### Feature selection and dataset split

Machine learning models were developed with the goal of predicting a patient-reported EQ-5D FHS at baseline and week 52. Feature selection was conducted using the Monte Carlo Feature Selection (MCFS) algorithm, applied through the rmcfs R package version 1.3.5 (R Foundation for Statistical Computing, Vienna, Austria). The MCFS was chosen due to its robustness and capacity to handle feature selection across multiple permutations. This approach was favoured over alternatives due to its ability to generate a prioritized list of relevant features and manage complex interdependencies. The only difference between the pools of variables input into the algorithm for the baseline and week 52 models was the inclusion of the baseline EQ-5D index scores in the algorithm for the latter models.

With the MCFS, a prioritized list of relevant features was generated and served as the determinants of the outcome. Selection of the optimal number of features for the models was conducted by assessing their relative importance from a ranked list, using the permutation method [24]. Next, further refinement of the feature selection was made based on collinearity and clinical relevance.

Patients with incomplete data on the selected features and outcome variables were removed from the datasets during subsequent analysis. Preprocessing steps included data normalization, conducted internally by the caret R package (see below) during training, ensuring features were scaled appropriately for algorithms like SVM and neural networks. Upon finalization of the datasets for the baseline and week 52 analysis, each dataset was randomly split into a training and a test set at a ratio of 4:1, respectively, using the caret R package version 6.0-94 [25]. Since there was a class skew in the outcome variable, oversampling was applied to the training set to address this issue, using the R package ROSE version 0.0-4 [26].

### Training and testing of the machine learning models

The following algorithms were employed: support vector machine with radial basis function kernel (SVMRadial), least absolute shrinkage and selection operator (LASSO), neural network (NNet), and logistic regression (LR). SVMRadial and NNet were selected for their capacity to model non-linear relationships, while LASSO and LR offered complementary insights into linear relationships with improved interpretability. Tree-based methods such as random forests or gradient boosting machines, although suitable for capturing feature interactions, were not employed, prioritizing model simplicity and interpretability given the dataset size and scope. Predictions were classified as positive (i.e. EQ-5D FHS experience) if their respective probabilities were ≥0.5. To prevent overfitting, hyperparameter tuning was automatically done through 10 times 10-fold cross-validation (CV). Both training and CV were conducted using the caret R package [25]. The optimal hyperparameters for the models were as follows: SVMRadial: σ = 0.2498819, C = 1 for baseline and σ = 0.1994953, C = 0.25 for week 52; LASSO: α = 1, λ = 0.002 for baseline and α = 1, λ = 0.04 for week 52; NNet: size = 5, decay = 0.1 for baseline and size = 1, decay = 0.1 for week 52.

After having obtained and trained the final models, these were evaluated using the test sets. The performance metrics encompassed accuracy, sensitivity, specificity, positive predictive value (PPV), negative predictive value (NPV), receiver operating characteristics (ROC) curves for illustrative purposes and area under the curve (AUC). The two latter performance measures were calculated using the pROC R package version 1.18.0 [27], while the others were assessed through the caret R package [25]. Finally, calibration curves were plotted and smoothed (resorting to the B-spline function) to determine the degree of alignment between predicted and observed outcome probabilities using the following Python libraries: pandas (version 1.5.3) [28], scikit-learn (version 1.2.1) calibration module [29], NumPy (version 1.23.5) [30], SciPy (version 1.10.0) interpolate module [31] and Matplotlib (version 3.7.0) pyplot module [32].

### Statistical analysis

For normal distributions, descriptive statistics are indicated as number (percentage) or mean (s.d.), while for non-normal distributions, data are reported as median [interquartile range (IQR)]. Descriptive statistics and comparisons between patients who experienced an EQ-5D FHS and patients who did not were conducted using the tableone R package version 0.13.2 [33]. Differences yielding *P*-values <0.05 were considered statistically significant.

All analyses were performed in RStudio version 2023.03.0 + 386 (R Foundation for Statistical Computing), with the exception of calibration curves, which were plotted and smoothed using Python version 3.10.9 (Python Software Foundation, Wilmington, DE, USA).

## Results

Table 1 details demographic and baseline clinical characteristics for the overall study population as well as comparisons between patients who reported an EQ-5D FHS and patients who did not at baseline and at week 52. An EQ-5D FHS was reported by 12.9% of the patients at baseline and by 23.1% at week 52. The FHS subgroup (both at baseline and week 52) had more patients of Asian ancestry, while the non-FHS subgroup had more women and patients of older age or higher baseline BMI, SELENA-SLEDAI PGA, SDI or cSLEDAI-2K scores and lower baseline urine protein:creatinine ratio (UPCR). Patients in the FHS subgroup at week 52 reported greater EQ-5D index scores at baseline.

**Table 1.** Patients’ demographic and baseline clinical features, comparing the EQ-5D FHS and non-FHS subgroups at baseline and at week 52.

Characteristics	Overall (*N *=* *1642)	Baseline			Week 52		
		FHS (*n *=* *212)	Non-FHS (*n *=* *1430)	*P*-value	FHS (*n *=* *380)	Non-FHS (*n *=* *1262)	*P*-value
Female sex, *n* (%)	1544 (94.0)	192 (90.6)	1352 (94.5)	**0.033**	347 (91.3)	1197 (94.8)	**0.015**
Ancestry, *n* (%)							
Asian	332 (20.2)	76 (35.8)	256 (17.9)	**<0.001**	114 (30.0)	218 (17.3)	**<0.001**
Black/African American	142 (8.6)	8 (3.8)	134 (9.4)	**0.010**	22 (5.8)	120 (9.5)	**0.031**
Indigenous American	383 (23.3)	59 (27.8)	324 (22.7)	0.115	101 (26.6)	282 (22.3)	0.101
White/Caucasian	785 (47.8)	69 (32.5)	716 (50.1)	**<0.001**	143 (37.6)	642 (50.9)	**<0.001**
SDI score ≥1, *n* (%)	694 (42.3)	70 (33.0)	624 (43.7)	**0.004**	111 (29.2)	583 (46.2)	**<0.001**
Age, years, mean (s.d.)	37.78 (11.52)	34.51 (11.36)	38.26 (11.47)	**<0.001**	34.19 (10.78)	38.86 (11.53)	**<0.001**
BMI, mean (s.d.)	25.55 (5.91)	23.97 (4.69)	25.78 (6.04)	**<0.001**	24.08 (4.85)	25.99 (6.13)	**<0.001**
EQ-5D index score, mean (s.d.)	0.74 (0.19)	1.00 (0.00)	0.70 (0.17)	NA	0.86 (0.15)	0.70 (0.18)	**<0.001**
SELENA-SLEDAI PGA, mean (s.d.)	1.42 (0.48)	1.26 (0.47)	1.45 (0.48)	**<0.001**	1.34 (0.49)	1.45 (0.48)	**<0.001**
SDI score^a^, mean (s.d.)	0.79 (1.24)	0.53 (1.05)	0.82 (1.26)	**0.001**	0.47 (0.95)	0.88 (1.30)	**<0.001**
SLEDAI-2K score, mean (s.d.)	9.94 (3.82)	9.46 (3.76)	10.01 (3.82)	0.051	9.67 (3.99)	10.02 (3.76)	0.127
cSLEDAI-2K score, mean (s.d.)	7.31 (3.63)	6.21 (3.65)	7.47 (3.60)	**<0.001**	6.66 (3.84)	7.51 (3.54)	**<0.001**
SLE disease duration, years, median (IQR)	4.43 (1.48–9.32)	5.05 (1.56–9.46)	4.38 (1.47–9.28)	0.892	5.84 (5.64)	6.56 (6.54)	0.054
UPCR (mg/mg), median (IQR)	0.15 (0.09–0.36)	0.17 (0.10–1.10)	0.14 (0.09–0.33)	**<0.001**	0.56 (0.93)	0.45 (0.91)	**0.046**

NA: not applicable.

*P*-values in bold are statistically significant (*P *<* *0.05).

a This variable is not normally distributed; however, values are presented as means since medians would not be informative about differences between subgroups.

### Feature selection

Results from the MCFS algorithm are presented in Supplementary Fig. S1, available at *Rheumatology Advances in Practice* online. The algorithm deemed 17 and 41 features informative for the baseline and week 52 models, respectively. Most of them were related to disease activity and organ damage. Other informative features included age, sex, Asian ancestry and baseline BMI, as well as baseline EQ-5D data for the week 52 models, which also constituted the most informative group of features for these models.

With the posterior refinement of feature selection, the following features were made final for both models: age, sex, Asian ancestry, baseline cSLEDAI-2K score, baseline SELENA-SLEDAI PGA and baseline UPCR. For the week 52 models, apart from these features, EQ-5D index scores were also included.

### Training and test sets

Each training set contained 80% (*n *=* *1314) of the total dataset and each test set contained the remaining 20% (*n *=* *328). No statistically significant differences were found for any of the final features between the training and test sets, at either time point (Supplementary Table S1, available at *Rheumatology Advances in Practice* online). This suggests a similar feature distribution and indicates that the models were trained on datasets that are representative of the data encountered during testing.

### Performance of the models

Regarding the training sets, the AUCs for the baseline SVMRadial, LASSO, NNet and LR models were 0.83, 0.73, 0.78 and 0.73, respectively. The corresponding values for the week 52 models were 0.83 for the SVMRadial model and 0.80 for the other three.

**Figure 1.**
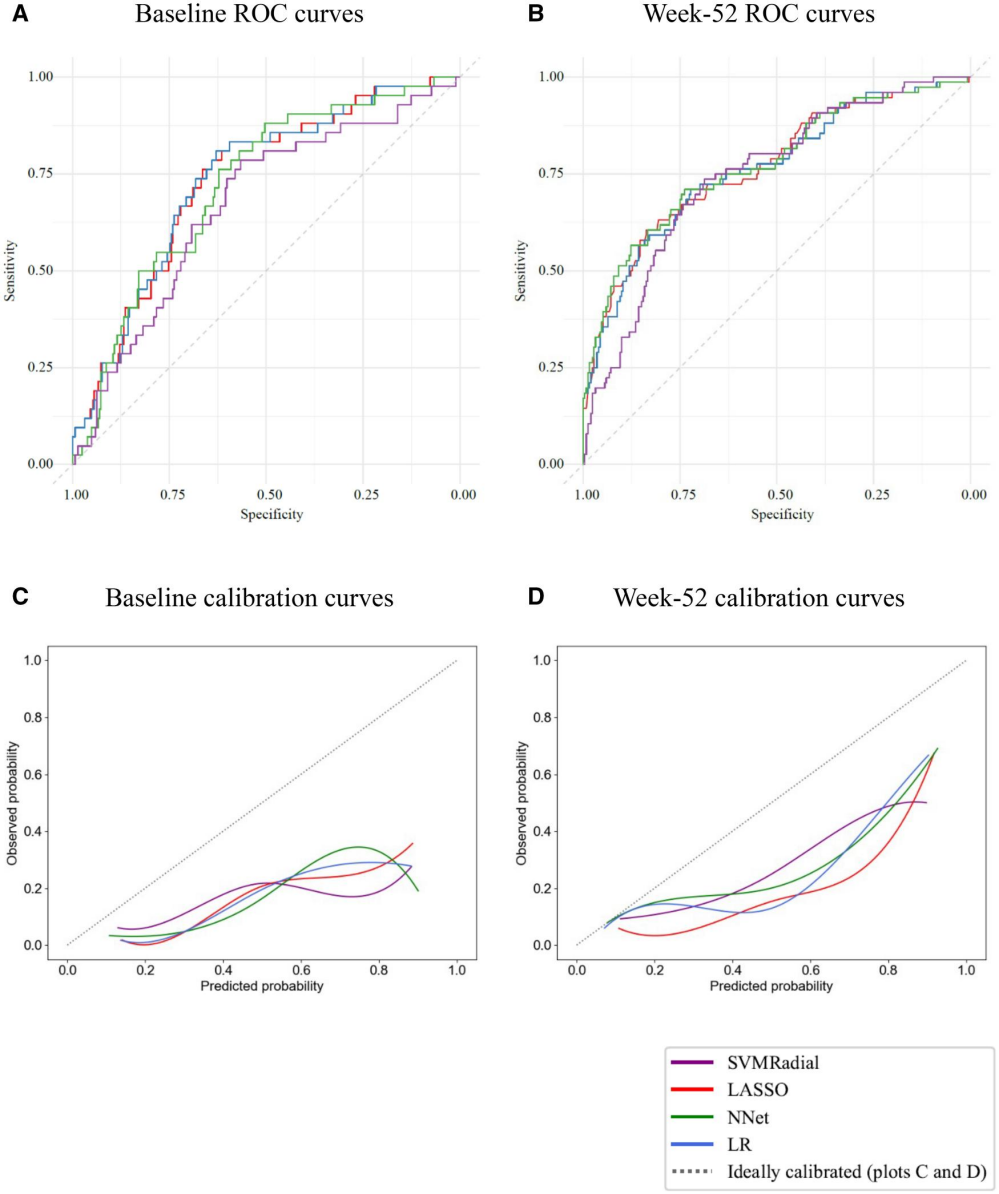
ROC curves on the test datasets from the **(A)** baseline and **(B)** week 52 models and smoothed calibration curves on the same datasets from the **(C)** baseline and **(D)** week 52 models

Regarding the test sets, the performance metrics for the baseline and week 52 models are presented in Table 2. Overall, the performance of the models was similar in the baseline and week 52 analyses. However, comparing the baseline models with the week 52 models, a slightly better performance was documented based on AUC metrics for the week 52 models, i.e. a maximum of 0.73 was seen for the baseline LASSO and LR models *vs* a maximum of 0.77 for the week 52 LASSO and NNet models. Additionally, the sensitivity and PPV were overall better in the week 52 models (sensitivity: 0.52–0.71 for baseline and 0.67–0.72 for week 52; PPV: 0.21–0.25 for baseline and 0.39–0.44 for week 52). Regarding NPVs, these were particularly high for all models (0.88–0.94). The ROC curves from the models at both time points are shown in Fig. 1, and the confusion matrices are presented in Supplementary Tables S2–S9, available at *Rheumatology Advances in Practice* online. Calibration curves, displayed in Fig. 1, show that all models exhibited poor calibration results at both time points, overestimating their predictions. Nevertheless, the week 52 models were slightly better calibrated, with greater predicted probabilities.

**Table 2.** Performance of baseline and week 52 models on the test sets.

Test	Baseline				Week 52			
	SVMRadial	LASSO	NNet	LR	SVMRadial	LASSO	NNet	LR
Accuracy (95% CI)	0.69 (0.64, 0.74)	0.69 (0.64, 0.74)	0.69 (0.63, 0.74)	0.69 (0.64, 0.74)	0.72 (0.67, 0.77)	0.67 (0.62, 0.72)	0.72 (0.67, 0.77)	0.69 (0.63, 0.74)
AUC	0.67	0.73	0.72	0.73	0.75	0.77	0.77	0.76
Sensitivity	0.52	0.71	0.55	0.69	0.67	0.72	0.71	0.72
Specificity	0.72	0.69	0.71	0.69	0.73	0.65	0.73	0.67
PPV	0.21	0.25	0.21	0.25	0.43	0.39	0.44	0.40
NPV	0.91	0.94	0.91	0.94	0.88	0.89	0.89	0.89

## Discussion

The main purpose of this post hoc analysis of the BLISS-52 and BLISS-76 trials was to identify factors that are associated with EQ-5D FHS reports at baseline and 52 weeks after commencement of a therapeutic intervention with belimumab or placebo on top of standard therapy, using machine learning algorithms. Following feature selection by means of the MCFS algorithm, mitigation was attempted based on collinearity and clinical relevance. The final models demonstrated that older age, female sex, non-Asian ancestry, high SELENA-SLEDAI PGA, high cSLEDAI-2K scores and low UPCR were negatively associated with an EQ-5D FHS experience both at baseline and after the trial intervention, while high baseline EQ-5D index scores constituted the most important determinant of an EQ-5D FHS experience at week 52.

Relating to sex, our findings are in line with previous studies demonstrating women with SLE report worse HRQoL compared with men [34–36]. The relationships between age or disease activity and HRQoL have not been consistent in previous SLE literature [34–39]; we herein found that advancing age and greater clinical disease activity were negatively associated with an EQ-5D FHS experience. Discrepancies in the literature may be multifactorial, from differences across populations to the large variation in instruments used to measure disease activity or estimate HRQoL. The most common instrument for estimating HRQoL has probably been the Medical Outcomes Study 36-item Short Form [39], with only a few studies hitherto having used EQ-5D in SLE populations [13, 38]. Despite the large interrater variability for PGA scoring, higher PGA scores have consistently been negatively associated with HRQoL in patients with SLE [40–42], which highlights that PGA captures features of disease activity beyond the span captured by the SLEDAI.

Although patients of Asian origin with SLE are known to have a more severe disease burden compared with White/Caucasian patients [43], we herein demonstrated that Asian patients more frequently reported an EQ-5D FHS compared with the non-Asian population. In fact, this is in line with previous literature that documented a tendency of Asian people with SLE to report better HRQoL [44], with social and cultural determinants influencing reporting trends likely underlying this observation.

There is limited information in previous studies regarding the relationship between renal involvement and SLE patients’ HRQoL. Daleboudt *et al*. [45] reported a moderate inverse correlation between proteinuria and HRQoL in a study population of proliferative LN, where substantial reductions in proteinuria were seen after successful treatment. Higher UPCR levels were herein associated with an EQ-5D FHS experience, both at baseline and week 52, which contrasts with the study by Daleboudt *et al*. [45] and is seemingly counterintuitive. Importantly, the BLISS trials excluded patients with active severe LN during the screening phase, resulting in relatively low proteinuria levels in the overall study population, thus limiting the generalizability of this finding to SLE populations with more active renal disease. Nonetheless, our observation contrasts with what one would consider an expected finding and is therefore of some interest, as it underscores that subclinical proteinuria is not sensed by SLE patients in a way that affects patient-reported HRQoL. This calls for attentive surveillance of renal manifestations with frequent urinalysis and creatinine levels in people with SLE.

Finally, baseline EQ-5D-3L index scores constituted the most important feature in the week 52 models, with greater scores predicting an EQ-5D FHS experience at week 52. This corroborates previous findings by means of logistic regression in a study by Lindblom *et al.* [13].

The selected machine learning algorithms were chosen to balance model performance with interpretability. While our study did not include tree-based methods such as random forests or gradient boosting machines, the use of neural networks and support vector machines with radial basis function kernels allowed for the capture of non-linear relationships in the data. This approach was deemed appropriate for the study objectives while avoiding the complexity and potential overfitting associated with tree-based methods, given the dataset size. Well-performing and easy-to-apply models for predicting an EQ-5D FHS experience in SLE would be useful, particularly since EQ-5D assessment has shown the ability to distinguish therapy responders from non-responders [13, 46] and FHS experience has been linked to a lower hazard to accrue organ damage [47]. Furthermore, the EQ-5D has demonstrated favourable psychometric properties in terms of validity and reliability [10], it is easy to fill (yielding a high rate of acceptability and completion) and it is simple to implement in clinical practice [48]. For all these reasons, the EQ-5D may be considered a useful tool for assessing HRQoL among currently used patient-reported outcome measures in SLE [46, 47], acknowledging that it provides important complementary information to current definitions of remission and low disease activity that solely rely on clinical and laboratory elements [49].

Regarding the performance of the machine learning models, no substantial differences were seen between AUCs deriving from the training and test sets, indicating an absence of overfitting issues. Moreover, AUCs from all models in the training sets were comprised of reasonable values, indicating the absence of underfitting as well. It is important to underscore that our models yielded very high NPVs, ≈90%, ensuring robust predictions of which patients are not likely to experience an EQ-5D FHS. In contrast, the downside of the models lies in the relatively low PPVs and the calibration curves, where a likely overestimation of predictions is noticed, with the week 52 models performing slightly better. This overestimation could stem from the class imbalance inherent in the dataset. Although oversampling using the ROSE package improved model training, it may have introduced synthetic data artifacts, limiting calibration accuracy. When executing oversampling, the algorithm randomly chooses examples from the minority class and replicates them, creating a balanced training set. This means that the positive examples (in our case the minority class) will yield a collection of repeated examples that does not provide sufficient information about the different possible distributions of features in positive examples. Moreover, if the oversampling technique does not adequately represent the variety of these examples in the original dataset (due to the randomness of example selection for replication), the models may not learn the nuanced patterns that are associated with the positive class.

The study has some limitations that should be acknowledged, including the post hoc nature of the analysis and the lack of data on comorbidities, socio-economic status and daily physical activity, features that are likely to contribute to SLE patients’ HRQoL experience. Limitations also included the lack of explicit statistical comparisons between model performances. Our focus was on identifying associated features rather than optimizing model performance. While the absence of an external validation dataset limits generalizability, our internal validation strategy ensured that the training and test sets were representative of the overall dataset. Additionally, while tree-based methods such as random forests or gradient boosting machines could provide further insights into feature interactions and complex relationships, our selected models offered a balanced approach between interpretability and performance. This approach aligns with our aim to focus on clinically interpretable outputs and avoid overfitting given the dataset size. We did not handle outliers—our analysis focused on utilizing the data as fully as possible without extensive cleaning. To mitigate the potential impact of outliers, secondary binary variables were included for some continuous features, which demonstrated consistency with the trends observed in the primary data. The low number of positive training examples (i.e. EQ-5D FHS reports) and the oversampling technique used to address this issue may have negatively affected the performance of our models, especially regarding the prediction of positive instances as explained above. Moreover, the generalizability of our findings is hindered by the exclusion of patients with active severe LN or neuropsychiatric lupus. Nonetheless, the study also has some important strengths, including the large and diverse SLE population and the extensive longitudinal data collection with a low degree of missing data, contributing to the robustness of findings. Another strength is the application of machine learning algorithms, which, to our knowledge, are used for the first time for the prediction of patient-reported outcomes, particularly the EQ-5D FHS experience.

Machado Escobar *et al.* [50] suggested that an EQ-5D index score ≥0.739 is indicative of good HRQoL among SLE patients, and a recent study by Hua *et al.* [46] advocated that an EQ-5D index score ≥0.800 distinguishes responders to treatment from non-responders. Thus an analysis focusing on predictive factors for achieving EQ-5D health states below FHS could offer valuable insights, as would study of determinants to differential responses across the different EQ-5D dimensions.

## Conclusions

Through a machine learning approach, we determined factors associated with an EQ-5D FHS experience at baseline and 52 weeks after the commencement of therapeutic intervention in a clinical trial setting. We found older age, female sex, non-Asian ancestry, high disease activity and low UPCR levels to be negatively associated with EQ-5D FHS reports. High baseline EQ-5D index scores constituted the most important predictor of an EQ-5D FHS at week 52. Our study contributes insights into determinants of patient-reported outcomes, a current major need towards person-centred care and integration of patient-reported health experiences in disease evaluation tools and treatment decisions.

## Supplementary Material

rkaf032_Supplementary_Data

## Data Availability

The datasets used and analysed in the present study can be made available through the Clinical Study Data Request consortium.
