# Supplementary material for: Predicting EQ-5D full health state in systemic lupus erythematosus using machine learning algorithms
Source: Rheumatol Adv Pract. 2025 Apr 18;9(2):rkaf032. doi: 10.1093/rap/rkaf032 (PMC12007597; doi:10.1093/rap/rkaf032)
Supplement: rkaf032_Supplementary_Data [file rkaf032_supplementary_data.zip › 24-153 Supplementary Material.docx]

**Supplementary Table S1.** Final features in the training and test sets (for the baseline and week-52 models).

|  |  | | |  | **Baseline** | | | | | | | | |  | **Week 52** | | | | | | | | |
| --- | --- | --- | --- | --- | --- | --- | --- | --- | --- | --- | --- | --- | --- | --- | --- | --- | --- | --- | --- | --- | --- | --- | --- |
|  | **Overall set**  (*n*=1642) | | |  | **Training set**  (*n*=1314) | | |  | **Test set**  (*n*=328) | | |  |  |  | **Training set**  (*n*=1314) | | |  | **Test set**  (*n*=328) | | |  |  |
|  |  |  |  |  |  |  |  |  |  |  |  |  |  |  |  |  |  |  |  |  |  |  |  |
|  | ***n*** | **(%)** |  | | ***n*** | **(%)** |  | | ***n*** | **(%)** |  | | ***p*-value** |  | ***n*** | **(%)** |  | | ***n*** | **(%)** |  | | ***p*-value** |
| **Female sex** | 1544 | (94.0) |  | | 1238 | (94.2) |  | | 306 | (93.3) |  | | 0.616 |  | 1242 | (94.5) |  | | 302 | (92.1) |  | | 0.123 |
| **Asian ancestry** | 332 | (20.2) |  | | 259 | (19.7) |  | | 73 | (22.3) |  | | 0.342 |  | 261 | (19.9) |  | | 71 | (21.6) |  | | 0.521 |
|  | **mean** | **(SD)** |  | | **mean** | **(SD)** |  | | **mean** | **(SD)** |  | |  |  | **mean** | **(SD)** |  | | **mean** | **(SD)** |  | |  |
| **Age**, years | 37.78 | (11.52) |  | | 37.97 | (11.47) |  | | 36.99 | (11.70) |  | | 0.168 |  | 37.59 | (11.45) |  | | 38.51 | (11.81) |  | | 0.196 |
| **EQ-5D utility index score** | 0.74 | (0.19) |  | | *NA* | *NA* |  | | *NA* | *NA* |  | | *NA* |  | 0.74 | (0.19) |  | | 0.75 | (0.19) |  | | 0.355 |
| **SELENA-SLEDAI PGA** | 1.42 | (0.48) |  | | 1.43 | (0.48) |  | | 1.40 | (0.49) |  | | 0.398 |  | 1.42 | (0.49) |  | | 1.44 | (0.47) |  | | 0.440 |
| **cSLEDAI-2K scores** | 7.31 | (3.63) |  | | 7.30 | (3.66) |  | | 7.35 | (3.51) |  | | 0.839 |  | 7.30 | (3.53) |  | | 7.35 | (4.01) |  | | 0.826 |
|  | **median** | **(IQR)** | |  | **median** | **(IQR)** | |  | **median** | **(IQR)** | |  |  |  | **median** | **(IQR)** | |  | **median** | **(IQR)** | |  |  |
| **UPCR**, mg/mg | 0.15 | (0.09–0.36) | |  | 0.14 | (0.09–0.36) | |  | 0.16 | (0.09–0.40) | |  | 0.438 |  | 0.15 | (0.09–0.38) | |  | 0.15 | (0.09–0.32) | |  | 0.934 |

cSLEDAI-2K: clinical Systemic Lupus Erythematosus Disease Activity Index 2000; IQR: interquartile range; NA: not applicable; PGA: Physician Global Assessment; SD: standard deviation; UPCR: urine protein to creatinine ratio.

**Supplementary Table S2.** Confusion matrix for the baseline SVMRadial model.

|  |  | **Observed number of patients** | |
| --- | --- | --- | --- |
|  |  | **FHS** | **Non-FHS** |
| **Predicted number of patients** | **FHS** | 22 | 81 |
|  | **Non-FHS** | 20 | 205 |

FHS: full health state; SVMRadial: support vector machine with radial basis function kernel.

**Supplementary Table S3.** Confusion matrix for the baseline LASSO model.

|  |  | **Observed number of patients** | |
| --- | --- | --- | --- |
|  |  | **FHS** | **Non-FHS** |
| **Predicted number of patients** | **FHS** | 30 | 90 |
|  | **Non-FHS** | 12 | 196 |

FHS: full health state; LASSO: least absolute shrinkage and selection operator.

**Supplementary Table S4.** Confusion matrix for the baseline NNet model.

|  |  | **Observed number of patients** | |
| --- | --- | --- | --- |
|  |  | **FHS** | **Non-FHS** |
| **Predicted number of patients** | **FHS** | 23 | 84 |
|  | **Non-FHS** | 19 | 202 |

FHS: full health state; NNet: neural network.

**Supplementary Table S5.** Confusion matrix for the baseline LR model.

|  |  | **Observed number of patients** | |
| --- | --- | --- | --- |
|  |  | **FHS** | **Non-FHS** |
| **Predicted number of patients** | **FHS** | 29 | 89 |
|  | **Non-FHS** | 13 | 197 |

FHS: full health state; LR: logistic regression.

**Supplementary Table S6.** Confusion matrix for the week-52 SVMRadial model.

|  |  | **Observed number of patients** | |
| --- | --- | --- | --- |
|  |  | **FHS** | **Non-FHS** |
| **Predicted number of patients** | **FHS** | 51 | 67 |
|  | **Non-FHS** | 25 | 185 |

FHS: full health state; SVMRadial: support vector machine with radial basis function kernel.

**Supplementary Table S7.** Confusion matrix for the week-52 LASSO model.

|  |  | **Observed number of patients** | |
| --- | --- | --- | --- |
|  |  | **FHS** | **Non-FHS** |
| **Predicted number of patients** | **FHS** | 55 | 87 |
|  | **Non-FHS** | 21 | 165 |

FHS: full health state; LASSO: least absolute shrinkage and selection operator.

**Supplementary Table S8.** Confusion matrix for the week-52 NNet model.

|  |  | **Observed number of patients** | |
| --- | --- | --- | --- |
|  |  | **FHS** | **Non-FHS** |
| **Predicted number of patients** | **FHS** | 54 | 69 |
|  | **Non-FHS** | 22 | 183 |

FHS: full health state; NNet: neural network.

|  |  | **Observed number of patients** | |
| --- | --- | --- | --- |
|  |  | **FHS** | **Non-FHS** |
| **Predicted number of patients** | **FHS** | 55 | 82 |
|  | **Non-FHS** | 21 | 170 |

**Supplementary Table S9.** Confusion matrix for the week-52 LR model.

FHS: full health state; LR: logistic regression.

**Supplementary Figure S1.** Monte Carlo Feature Selection (MCFS) plots for baseline (**A**) and week 52 (**B**). ALP: alkaline phosphatase; ALT: alanine transaminase; ANA: antinuclear antibody; aPTT: activated partial thromboplastin time; AST: aspartate transferase; BILAG: British Isles Lupus Assessment Group; BMI: body mass index; BUN: blood urea nitrogen; C3: complement component 3; C4: complement component 4; SLEDAI-2K: clinical Systemic Lupus Erythematosus Disease Activity Index 2000; eGFR: estimated glomerular filtration rate FFS: full health state; LLDAS: Lupus Low Disease Activity State; PGA: Physician Global Assessment; SDI: Systemic Lupus International Collaborating Clinics (SLICC)/American College of Rheumatology (ACR) Damage Index; SELENA-SLEDAI: Safety of Estrogens in Lupus National Assessment – Systemic Lupus Erythematosus Disease Activity Index; SLE: systemic lupus erythematosus; SLEDAI-2K: Systemic Lupus Erythematosus Disease Activity Index 2000; UPCR: urinary protein to creatinine ratio; VAS: visual analogue scale.
